# The Life and Growth of the Tooth

**Published:** 1896-12

**Authors:** D. D. Smith


ARTICLE IV.

### THE LIFE AND GROWTH OF THE TOOTH.

DR. D. D. SMITH.

The full apprehension of the fact that the different formations which make up the body of the tooth receive life and nourishment from two distinct sources, the pulp and the pericementum, and of the phenomena arising from it, will greatly assist in establishing rational and substantial methods for the treatment and saving of teeth. The pulp, which is made up of nerve-tissue and of vessels for the supply of nutriment and for taking up waste, is the important factor of every tooth. To it is committed the care of the newly-erupted tooth, to readjust, recalcify, consolidate, strengthen and sustain the enamel and dentine. Hence the importance of keeping the pulps of young teeth in a condition of healthful activity, carefully guarding them against any encroachment through decay or manipulation. The teeth in mastication, for children, is of more importance than inheritance as a factor in determining the character of the teeth. Natural and healthful mastication, the true cleaner of the teeth, would alone force that exercise required by the pulp and peridentum. Devitalization of the pulp at an early period of life carries with it retrogressive changes in the quality of tooth material without power to arrest it.

So general is the action of the pulp in rearranging. depositing and solidifying the materials of the dentine and enamel of young teeth, that a tooth, fragile and imperfect at eruption, if introduced into conditions of use and cleanliness, and the pulp protected, may be built into an organ of good type, practically decay-resisting. Isolated instances of this have been observed, but are regarded as exceptional rather than as the true expression of pulp-action. Are not the teachings of the past and present wanting in giving the position of supreme importance to operations on the externals of the tooth, and would it not be more in harmony with the true science of tooth-building to emphasize pulp protection in young life.—*Cosmos.*

